# The presence of diabetic retinopathy closely associated with the progression of non-alcoholic fatty liver disease: A meta-analysis of observational studies

**DOI:** 10.3389/fmolb.2022.1019899

**Published:** 2022-11-15

**Authors:** Guo-heng Zhang, Tian-hao Yuan, Zhen-sheng Yue, Lin Wang, Guo-Rui Dou

**Affiliations:** ^1^ Department of Ophthalmology, Eye Institute of Chinese PLA, Xijing Hospital, Fourth Military Medical University, Xi’an, China; ^2^ Department of Ophthalmology, 942 Hospital of the Joint Logistics Support Force of the Chinese People’s Liberation Army, Yin’chuan, China; ^3^ Department of The Cadet Team 6 of School of Basic Medicine, Fourth Military Medical University, Xi’an, China; ^4^ Department of Hepatobiliary Surgery, Xijing Hospital, Fourth Military Medical University, Xi’an, China

**Keywords:** non-alcoholic fatty liver disease, diabetes, diabetic retinopathy, liver fibrosis, meta-analysis

## Abstract

**Background and Objective:** Although growing evidence indicates that non-alcoholic fatty liver disease is related to diabetic retinopathy (DR), research results significantly vary. Therefore, we conducted a meta-analysis to assess the association between the progression of non-alcoholic fatty liver disease and the onset of DR.

**Methods:** PubMed, Embase, and Cochrane databases were searched until 7 November 2021. Combined odds ratios (ORs) and 95% confidence intervals (CIs) were used to assess the association.

**Results:** We identified 18 studies involving 12,757 patients. The pooled effect assessment showed that liver fibrosis was positively correlated with DR (OR = 1.69, 95%CI 1.30–2.20; *p* < 0.0001); non-alcoholic fatty liver disease was not associated with the risk of DR (OR = 1.15, 95%CI 0.75-1.76; *p* = 0.51); non-alcoholic fatty liver disease was positively correlated with DR in patients with type 1 diabetes (OR = 2.96, 95%CI 1.48–5.94; *p* = 0.002). In patients with type 2 diabetes, there was no association between non-alcoholic fatty liver disease and DR (OR = 0.92, 95%CI 0.59–1.43; *p* = 0.70). Subgroup analysis showed no correlation in both Asian and Caucasian races.

**Conclusion:** There is a significant correlation between liver fibrosis and DR. This suggests that the ocular examination of DR could be helpful in predicting whether patients with non-alcoholic fatty liver disease would progress to liver fibrosis.

## 1 Introduction

Non-alcoholic simple fatty liver, non-alcoholic steatohepatitis, and cirrhosis are a series of progressive chronic liver diseases (Farrell and Larter, 2006). They are generally referred to as non-alcoholic fatty liver disease (NAFLD), with a global prevalence of up to 25% (Younossi et al., 2016). As overall mortality, liver-specific morbidity, and mortality are significantly increased in NAFLD patients with evidence of nonalcoholic steatohepatitis and advanced fibrosis, the early screening of these subsets of patients is of vital clinical significance (Cotter and Rinella, 2020). The current gold standard for NAFLD diagnosis is liver biopsy, but there are limitations such as its sampling bias, poor acceptability, and serious complications (Zhou et al., 2019). Ultrasound and magnetic resonance imaging (MRI) of liver fat can be used to diagnose NAFLD. Importantly, ultrasound and MRI have limited sensitivity in the diagnosis of fatty liver when the degree of hepatocyte steatosis is not high (Eslam et al., 2020). When NAFLD progresses to a more severe stage with liver fibrosis, NAFLD fibrosis score (NFS) and transient elastography measurement of liver stiffness (LSM) can benefit the diagnosis. Their effects have been confirmed in some studies (Newsome et al., 2020; Lee et al., 2021). Hence, more non-invasive diagnostic methods or biomarkers are to be explored to assist in the monitoring of NAFLD progression.

There is a general consensus that diabetes and NAFLD exert synergistic effects. Impairment of glucose and lipid metabolic pathways, which is caused by the worldwide increase in the prevalence of obesity and type 2 diabetes mellitus (T2DM), is most likely behind the increase in people with NAFLD (Smith et al., 2020; Stefan and Cusi, 2022). Diabetes is regarded to increase the risk of NAFLD progression to non-alcoholic steatohepatitis (NASH), cirrhosis, and hepatocellular carcinoma (Armandi and Schattenberg, 2021; Khandelwal et al., 2021; Chen et al., 2022), whereas NAFLD can increase the incidence of complications in diabetic patients, especially vascular complications (Hazlehurst et al., 2016; Perumpail et al., 2017). However, a relation between the presence and severity of NAFLD and the development and progression of micro and macrovascular complications in diabetes is still a subject of debate. Diabetic retinopathy (DR), as one of the most common microvascular complications of DM, has also been connected to NAFLD, but the conclusions are controversial (Yuan et al., 2021). A recent study has suggested that there was no association between NAFLD and DR in individuals with T2DM (Song et al., 2021). However, a significant association between liver fibrosis and the presence of microvascular complications has been recently reported, indicating a potential link between NAFLD severity and DR onset. As DR pathogenesis involving metabolic disturbance and features of insulin resistance shows a high degree of overlap with the mechanisms of NAFLD, it is rational that in a population at high risk for DR, such as the diabetic population, the presence of advanced liver fibrosis may further increase this risk. As ocular signs have been more and more referred to in the diagnosis and progression in the nervous system, cardiovascular, and liver diseases (Gubitosi-Klug et al., 2021; Yuan et al., 2021; Cunha et al., 2022), it is intriguing that a simple non-invasive screening of DR, detected by using a routine ocular check-up, may allow the detection of patients with T2DM at risk of advanced NAFLD. Thus, clarifying the correlation between DR and the onset and progression of NAFLD will be a great help in defining if DR can be a non-invasive indicator in monitoring NAFLD progression.

Based on the above, we performed this meta-analysis to assess the association between DR and NAFLD to have a clearer and deeper understanding of the correlation between them and to provide new ideas for the screening and monitoring of NAFLD.

## 2 Materials and methods

The study was conducted in adherence to the Preferred Reporting Items for Systematic Reviews and Meta-Analyses (PRISMA) Statement (Moher et al., 2009). A checklist is included in Appendix A (PRISMA 2009 checklist).

### 2.1 Search strategy

We conducted a detailed search using “NAFLD”, “NASH”, “DR”, “liver fibrosis”, and related keywords. The detailed retrieval strategy is given in Supplementary Table S1. As of November 2021, we searched the following databases: PubMed, Cochrane, and Embase. Furthermore, we imported the search results into Endnote (version X9.3.3) for merging.

### 2.2 Study selection and inclusion criteria

Two researchers (TH Yuan and GH Zhang) independently screened the eligibility of retrieved articles. The inclusion criteria we adopted were as follows: (1) cross-sectional, cohort, or prospective studies in humans; (2) evaluation the association between NAFLD or liver fibrosis and DR in the study; (3) evaluation of liver steatosis by liver imaging (ultrasound, computed tomography, magnetic resonance imaging, or spectroscopy) or liver biopsy as diagnostic criteria for NAFLD. The criteria for the diagnosis of NAFLD by imaging modalities are hepatocyte steatosis greater than 5% with/without inflammation and hepatocyte ballooning with/without liver fibrosis. The standard for steatosis evaluation does not include alcohol intake [≤140 g per week for women, ≤ 210 g per week for men (Sanyal et al., 2011)], viral hepatitis infection coexisting with liver disease, and using drugs known to cause hepatotoxicity and rapid weight loss. (4) Liver fibrosis is diagnosed by non-invasive liver fibrosis score (NFS) or liver stiffness measurement (LSM) in the population of patients with NAFLD. The value of LSM is ≥ 6.2 kPa by FibroScan^®^ examination (Echosens, Paris, France) (Eddowes et al., 2019; Newsome et al., 2020). The NFS formula includes age, body mass index (BMI), presence of diabetes or impaired fasting glycemia, and serum levels of AST, alanine aminotransferase (ALT), albumin and platelets (Angulo et al., 2007; Lee et al., 2021). (5) DR is diagnosed by fundus photography. According to the Early Treatment Diabetic Retinopathy Study (EDTRS), the diagnostic criteria for DR are retinal microhemangioma, retinal hemorrhage, exudation, or neovascularization (Early Treatment Diabetic Retinopathy Study Research Group, 2020). When a dispute arose, it was resolved through a joint reassessment with another researcher (GR Dou).

### 2.3 Quality assessment

The quality of each study was assessed independently by two researchers (GH Zhang and TH Yuan) using Agency for Healthcare Research and Quality (AHRQ). Based on three items, the quality of each article ranged from 1 to 11 stars. If there was any discrepancy, it was resolved through a joint reassessment with another researcher (GR Dou).

### 2.4 Data extraction

Two researchers (GH Zhang and TH Yuan) extracted data from each included article independently. The extracted data included the first author, year and country of publication, study design, sample size (male percentage), age of the sample population, type of diabetes, duration of diabetes, glycosylated hemoglobin (HbA1c), BMI, diagnostic criteria for NAFLD, the proportion of patients with NAFLD or DR, and the proportion of patients with liver fibrosis. If differences appeared, two researchers reached a consensus or negotiated with another author (GR Dou) to solve the problem.

### 2.5 Statistical analysis and heterogeneity test

The effect size (ES) was calculated based on the number of DR events (cases) in the NAFLD and non-NAFLD groups and the sample size of each study. The ES was reported as an odds ratio (OR) with a 95% confidence interval (CI). In the current meta-analysis, we used both fixed and randomized models. This approach was chosen because, in most cases, fixed models are used when studies are determined to be similar. However, when heterogeneity is classified as medium to high, the random-effects model is strongly recommended. The heterogeneity of the included studies was assessed using CHI^2^ and I^2^ tests. Heterogeneity was defined as high when I^2^ was greater than 75%, medium when I^2^ was between 50% and 75%, low when I^2^ was between 25% and 50%, and none when I^2^ was below 25%. Graphical evaluation of funnel plot and Egger regression asymmetry test were used to estimate potential publication bias. We conducted a further subgroup analysis to explore the causes of heterogeneity. Additionally, a sensitivity analysis was performed to eliminate the study one by one to observe their impact on the final effect evaluation. Data extracted from each study were processed and analyzed using Revman 5.4 and Stata 12.0.

## 3 Results

### 3.1 Selection results

**FIGURE 1:**
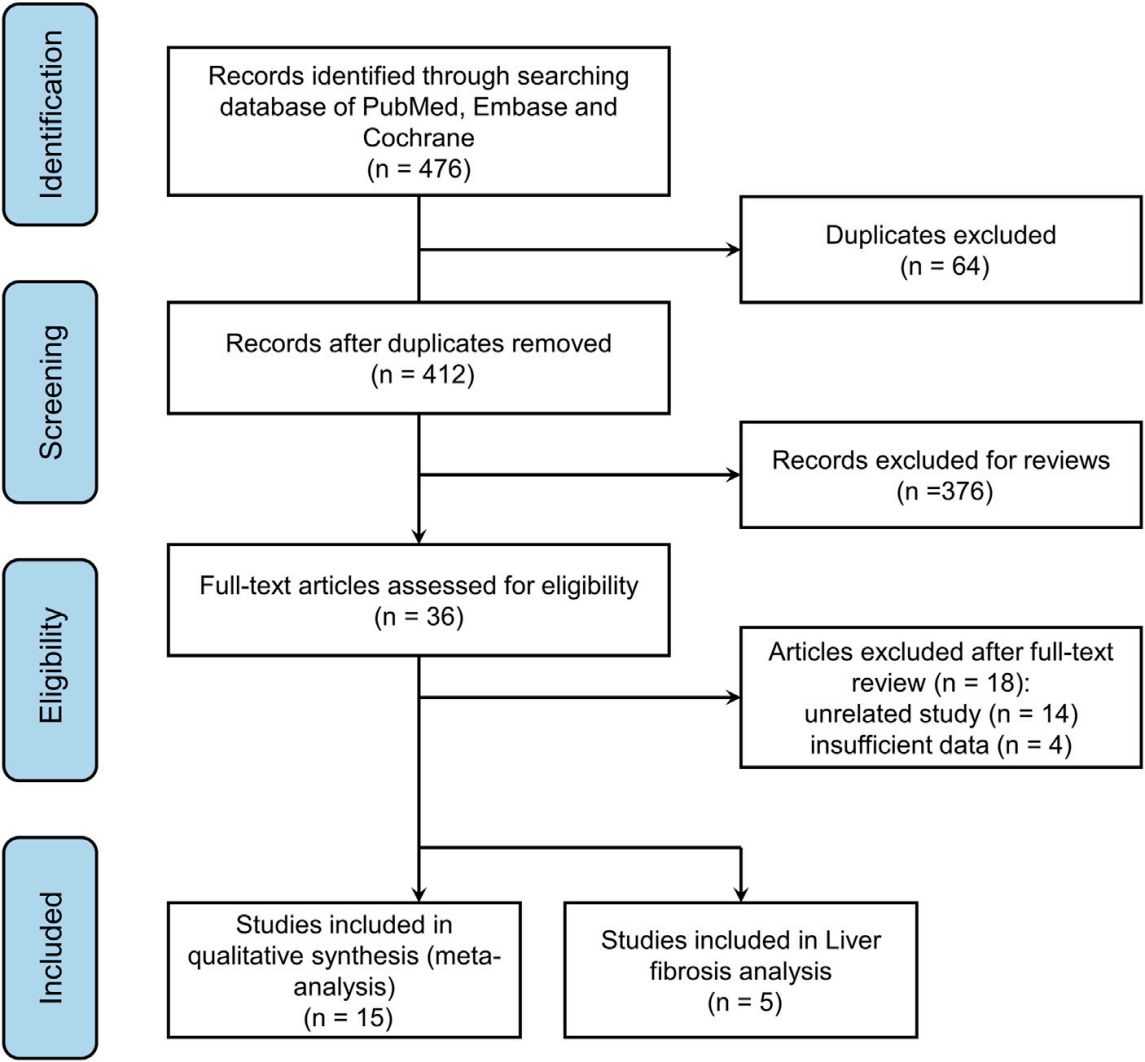
Flow chart of literature selection.

A total of 476 articles were obtained, and 412 articles were obtained after excluding duplicate literature. By reading the titles and abstracts, 376 articles were excluded. After reading the full text, 36 articles were screened out. Excluding 18 non-randomized controlled trials and outcome indicators, 18 studies were finally included in this meta-analysis. Fifteen of the 18 studies evaluated the association between NAFLD and DR (Targher et al., 2008; Targher et al., 2010a; Yoneda et al., 2012; Lv et al., 2013; Yang et al., 2013; Kim et al., 2014; Somalwar and Raut, 2014; Vendhan et al., 2014; Lin et al., 2016; Yan et al., 2016; Afarideh et al., 2019; Zhang et al., 2019; Lombardi et al., 2020a; Mikolasevic et al., 2021; Wen et al., 2021), five studies evaluated the association between liver fibrosis and DR in patients with NAFLD (Lombardi et al., 2020a; Lombardi et al., 2020b; Sawadjaan Leila and Ong Janus, 2020; Leite et al., 2021; Mikolasevic et al., 2021), and two of these studies included both of the above-mentioned research objectives (Lombardi et al., 2020a; Mikolasevic et al., 2021). The details of identifying qualified research and the exclusion criteria are given in Figure 1.

### 3.2 Basic characteristics and quality assessment

The main features of the 18 articles included are shown in Table 1, Table 2. Overall, 18 included articles involved 12,757 patients with diabetes (856 cases of type 1 diabetes [T1DM], 11,817 cases of T2DM, and 84 cases of unreported diabetes). Fifteen studies with the goal of diagnosing the incidence of DR in NAFLD involved 11,161 diabetic patients (6,199 with NAFLD and 4,962 without NAFLD), and five studies aimed at assessing the relationship between liver fibrosis and DR in NAFLD included 1,596 diabetic patients (476 with liver fibrosis, 1,120 without evidence of liver fibrosis). A total of 4036 DR events were included according to the clinical management guidelines for DR. Seventeen of these studies were cross-sectional, and one study was prospective. For quality assessment, we used the AHRQ (Henriksen et al., 2017), and the scores showed that all 18 studies were of high quality.

**TABLE 1:** Basic characteristics of the studies included in the review of association between non-alcoholic fatty liver disease and the risk of diabetic retinopathy in diabetes mellitus.

Author [Reference]	Year	Country	Sample size (% male)	Type of diabetic	NAFLD patients (%)	DR patients (%)	Age (years)	HbA1c (%)	Duration of DM (years)	BMI (kg/m^2^)	AST (U/L)	ALT (U/L)	Study quality
Targher et al. (2008)	2008	Italy	2,103 (61.70)	2	67.60	46.40	58.35	7.10	13.35	26.68	21.38	23.38	9
Targher et al. (2010a)	2010	Italy	202 (52.60)	1	54.90	38.10	42.50	8.22	18.40	24.75	22.75	24.95	9
Yoneda et al. (2012)	2012	Japan	144 (40.30)	1	17.40	12.50	48.96	7.58	14.92	22.77	21.82	20.11	8
Yang et al. (2013)	2013	China	825	2	31.60	20.00	NR	NR	NR	NR	NR	NR	5
Lv et al., (2013)	2013	China	1,217 (37.80)	2	61.00	45.80	63.39	8.73	9.58	26.26	20.74	21.97	9
Vendhan et al. (2014)	2014	India	510 (52.20)	1	31.20	40.40	19.39	10.17	7.55	24.88	27.70	35.04	8
Somalwar and Raut. (2014)	2014	India	120 (55.20)	2	31.20	40.40	55.20	7.36	9.83	25.24	29.84	32.99	8
Kim et al., (2014)	2014	Korea	929 (52.80)	2	63.30	46.70	57.65	8.36	6.19	24.88	27.70	35.70	9
Lin et al. (2016)	2016	US	945 (45.90)	2	48.60	20.50	57.93	7.56	NR	NR	NR	NR	8
Yan et al. (2016)	2016	China	212 (56.60)	2	67.50	37.70	53.67	9.10	7.49	26.89	21.44	19.24	7
Zhang et al. (2019)	2019	China	411 (47.40)	2	60.80	40.90	58.28	8.86	12.19	25.67	22.22	25.58	7
Afarideh et al. (2019)	2019	Iran	935 (48.10)	2	26.50	15.10	57.60	7.80	8.10	29.00	27.60	NR	8
Lombardi et al. (2020a)	2020	Italy	238 (52.00)	2	71.80	16.00	68.00	7.00	12.00	29.00	20.00	20.00	9
Mikolasevic et al. (2021)	2021	China	1928 (52.70)	2	61.30	23.20	57.00	9.30	10.00	26.03	18.00	18.00	9
Wen et al. (2021)	2021	Croatia	442 (47.30)	2	84.20	26.50	62.00	6.90	10.00	30.70	22.00	24.00	9

**TABLE 2:** Basic characteristics of the studies included in the review of association between liver fibrosis and the risk of diabetic retinopathy in diabetes mellitus.

Author [Reference]	Year	Country	Sample size (% male)	Type of diabetic	Live fibrosis patients (%)	DR patients (%)	Age (years)	HbA1c (%)	Duration of DM (years)	BMI (kg/m^2^)	AST (U/L)	ALT (U/L)	Study quality
Sawadjaan Leila and Ong Janus. (2020)	2020	Philippines	84 (58.90)	NR	46.40	8.30	56.12	NR	NR	29.01	NR	NR	5
Lombardi et al. (2020a)	2020	Italy	394 (52.00)	2	21.10	16.00	68.00	7.00	12.00	29.00	20.00	20.00	9
Lombardi et al. (2020b)	2020	Italy	351 (46.10)	2	21.90	16.20	68.31	6.90	12.33	28.86	NR	NR	8
Leite et al. (2021)	2021	Brazil	325 (37.70)	2	21.80	36.60	62.38	7.87	8.66	28.78	39.91	23.53	10
Mikolasevic et al. (2021)	2021	Croatia	442 (47.30)	2	46.60	26.50	62.00	6.90	10.00	30.70	22.00	24.00	9

### 3.3 NAFLD and DR risk

**FIGURE 2:**
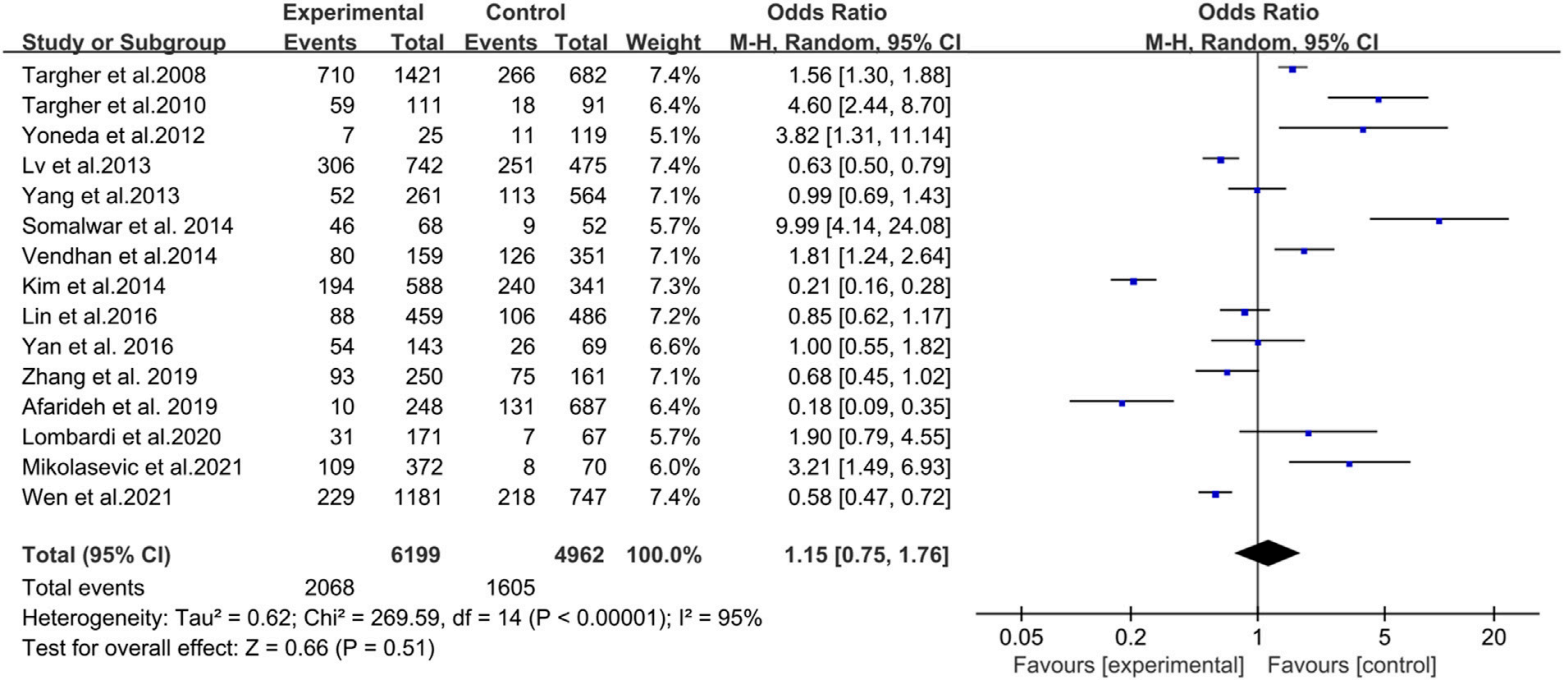
Meta-analysis of association between non-alcoholic fatty liver disease and the risk of diabetic retinopathy in diabetes mellitus.

In a pooled analysis of the 15 included studies, there was no association between NAFLD and DR in diabetic patients (OR = 1.15, 95% CI 0.75–1.76; *p* = 0.51) (Figure 2). However, the heterogeneity test showed that I^2^ = 95% (χ^2^ = 269.59, *p* < 0.001), indicating significant heterogeneity; thus, this study used the random effect model to draw a more conservative conclusion. Furthermore, the large heterogeneity suggested that we should view the results dialectically and conduct further in-depth research and analysis.

### 3.4 Liver fibrosis and DR risk

**FIGURE 3:**
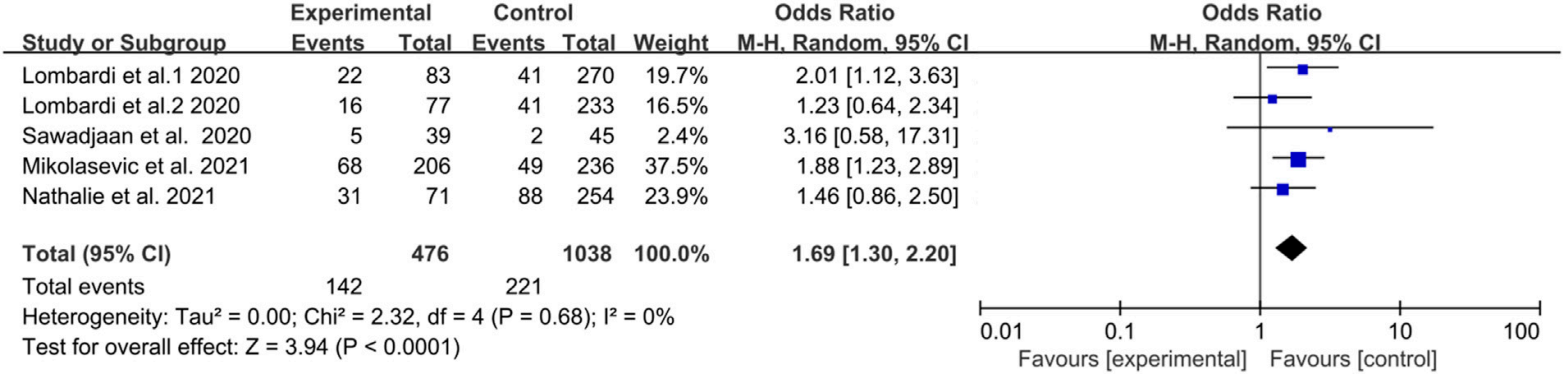
Meta-analysis of association between liver fibrosis and the risk of diabetic retinopathy in diabetes mellitus.

Five studies assessed the association between liver fibrosis and DR in diabetic patients. Overall, this analysis showed a positive association between liver fibrosis and DR in patients with diabetes (OR = 1.69, 95% CI 1.30–2.20; *p* < 0.0001) (Figure 3). Heterogeneity test showed I^2^ = 0% (χ^2^ = 2.32, *p* < 0.680), indicating that there was almost no heterogeneity. The random effects model was used to reach more conservative conclusions.

### 3.5 NAFLD and DR risk in T1DM

**FIGURE 4:**
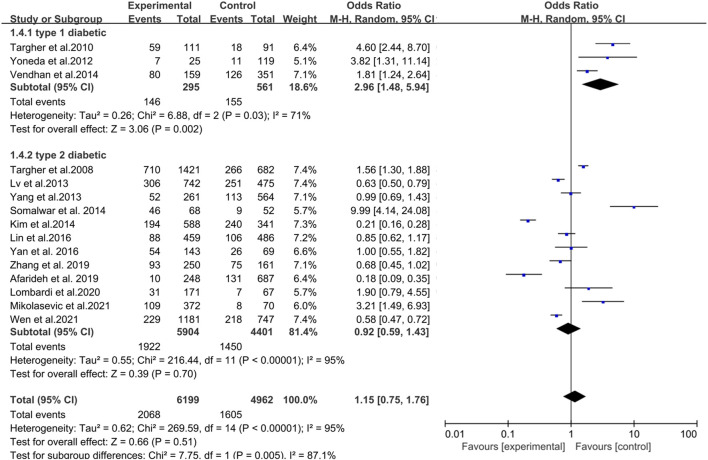
Meta-analysis of the association between nonalcoholic fatty liver disease and the risk of diabetic retinopathy in patients with different types of diabetes.

Three articles explored the effect of NAFLD and the incidence of DR in patients with T1DM. Their analysis showed a positive association between NAFLD and DR in patients with T1DM (OR = 2.96, 95% CI 1.48–5.94; *p* = 0.002) (Figure 4). However, the heterogeneity test showed I^2^ = 71% (χ^2^ = 6.88, *p* = 0.030), indicating moderate heterogeneity.

### 3.6 NAFLD and DR risk inT2DM

Twelve studies investigated the impact of NAFLD and the incidence of DR in patients with T2DM. Their analysis showed no statistical significance between NAFLD and DR in patients with T2DM (OR = 0.92, 95% CI 0.59–1.43; *p* = 0.70) (Figure 4). However, the heterogeneity test showed I^2^ = 95% (χ^2^ = 216.44, *p* < 0.001), indicating considerable heterogeneity.

### 3.7 Subgroup analyses

**FIGURE 5:**
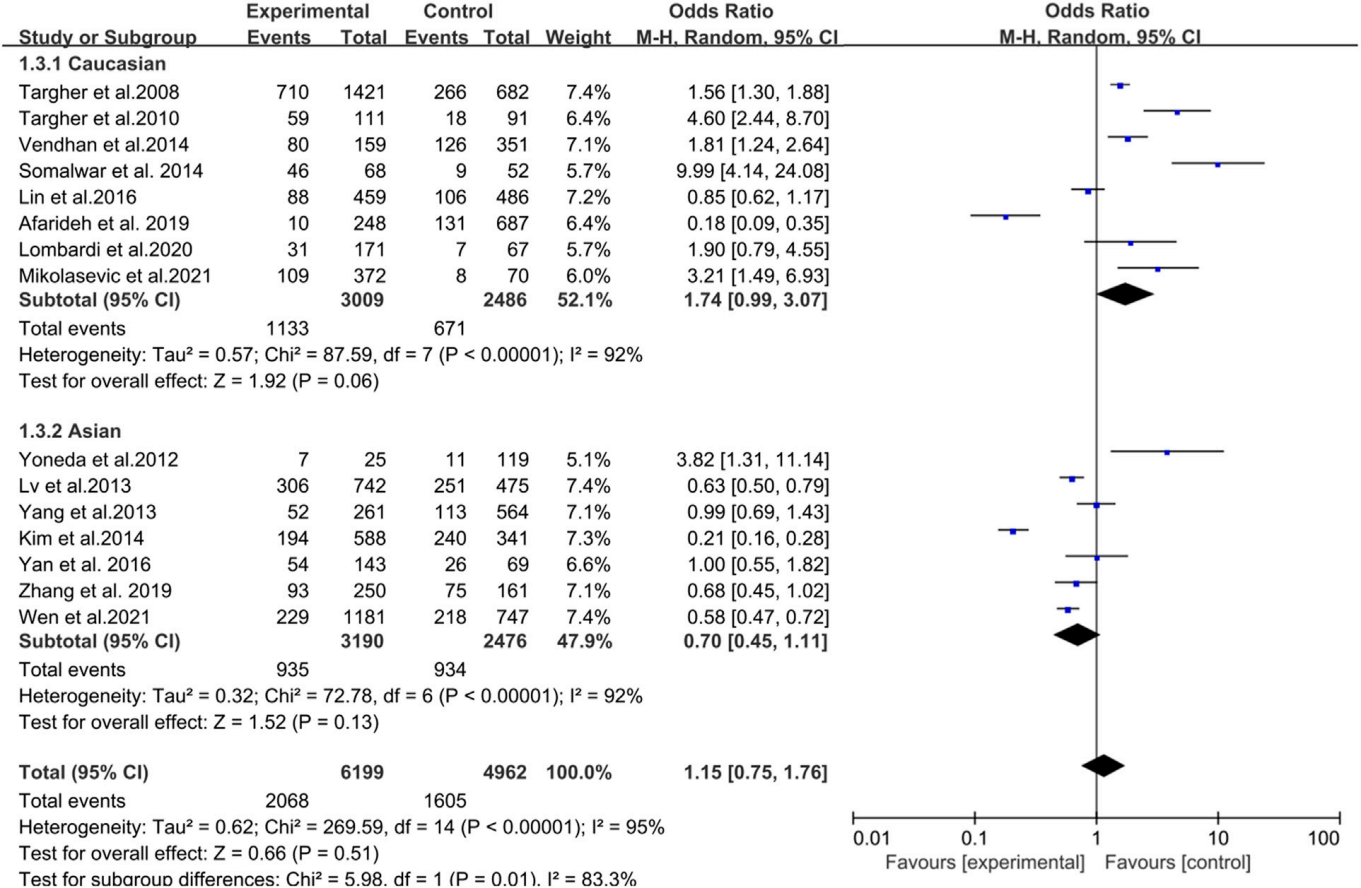
Meta-analysis of association between non-alcoholic fatty liver disease and the risk of diabetic retinopathy in diabetes mellitus based on the different races.

Groups were analyzed according to the ethnicity of the participants, including Caucasian and Asian races. As shown in Figure 5, in Caucasian ethnicity, diabetic patients with NAFLD had an increased risk of DR compared with patients without NAFLD, although the risk was not statistically significant (OR = 1.74, 95% CI: 0.99–3.07; *p* = 0.06). Heterogeneity tests showed that the results were highly heterogeneous (I^2^ = 91%, χ^2^ = 87.59, *p* < 0.001). Similarly, NAFLD was not associated with DR incidence in the Asian population (OR = 0.70, 95% CI: 0.45–1.11; *p* = 0.13). Heterogeneity tests showed that the results were highly heterogeneous (I^2^ = 91%, χ^2^ = 72.78, *p* < 0.001). Additionally, meta-regression analysis based on the mean age, course of diabetes, ALT, BMI, and HbA1c revealed no source of heterogeneity (Supplementary Table S2).

### 3.8 Sensitivity analysis and publication bias

**FIGURE 6:**
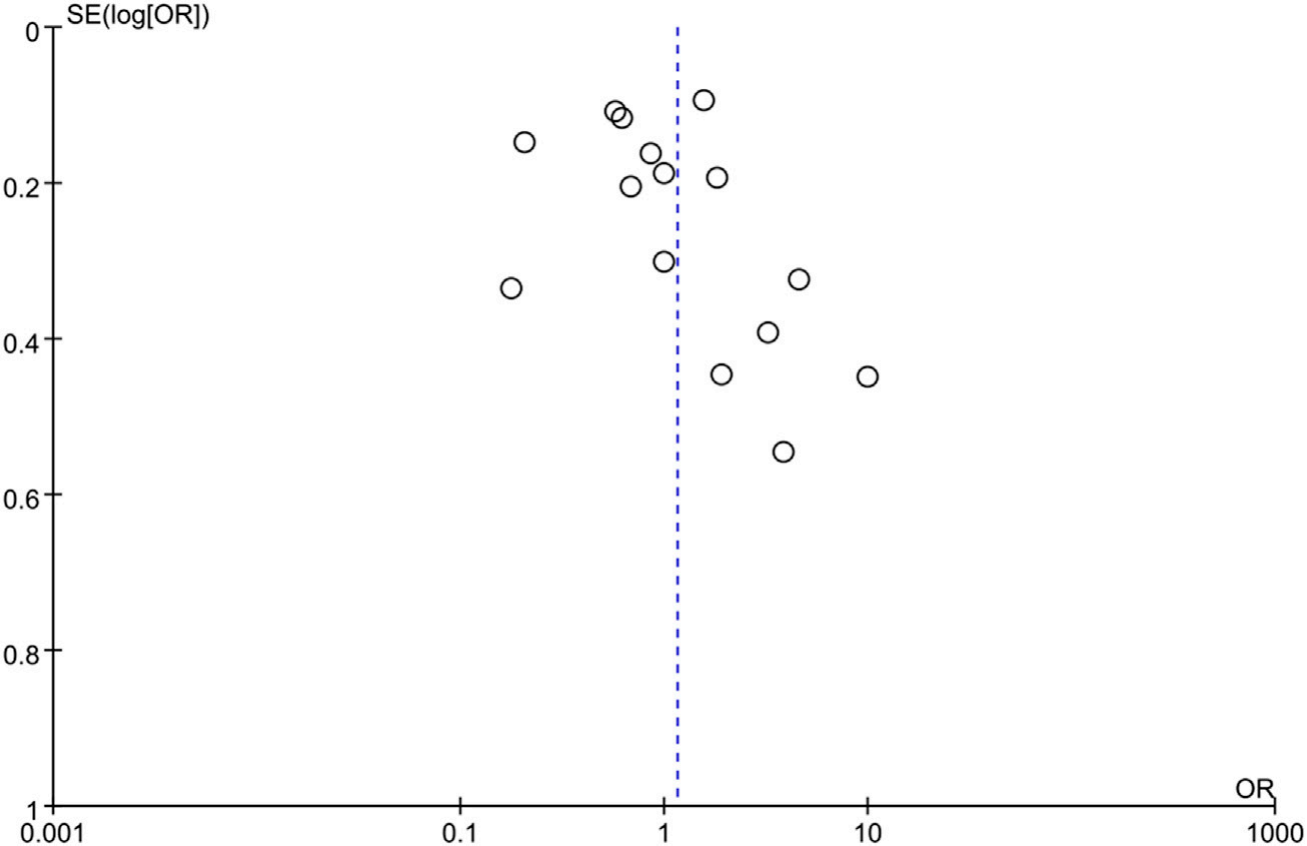
Funnel plots for meta-analysis of association between non-alcoholic fatty liver disease and the risk of diabetic retinopathy in diabetes mellitus.

In addition to subgroup analysis, sensitivity analysis was performed to explain the high heterogeneity of the results. Deletion of one study at a time showed no significant change (Supplementary Figures S1, S2). The funnel plot and the Begg funnel plots were roughly symmetrical, indicating no significant publication bias. (Figure 6, Supplementary Figures S3–S5).

## 4 Discussion

In this meta-analysis, 11,161 diabetic patients (6,199 with NAFLD and 4,962 without NAFLD) were selected to investigate the association between NAFLD and DR. To the best of our knowledge, compared with current systematic reviews and meta-analyses, our meta-analysis included the highest number of studies, conducted more subgroup analyses, and additionally explored the relationship between liver fibrosis and DR. Our study found a positive correlation between liver fibrosis and DR (OR = 1.69, 95% CI: 1.30–2.20), which suggested that the presence of DR might reflect the progression of liver fibrosis in NAFLD patients; hence, DR might be a predictor for progression to liver fibrosis in NAFLD patients. Patients with T2DM and severe liver fibrosis had a significantly higher risk of chronic vascular complications of diabetes (Hazlehurst et al., 2016). Furthermore, we found that the T1DM subgroup was positively associated with NAFLD in the subgroup analysis. Moreover, NAFLD was positively correlated with the risk of DR in Caucasian diabetic patients (OR = 1.74, 95% CI: 0.99–3.07). In contrast, NAFLD was negatively correlated with the risk of DR in Asian diabetic patients (OR = 0.70, 95% CI: 0.45–1.11), although the risk was not statistically significant.

The subgroup analysis above showed some positive results, but when they were put back into the whole analysis, something different emerged, and the association between DR and NAFLD disappeared. This was consistent with the findings of a recent meta-analysis exploring the relationship between NAFLD and DR in T2DM patients (Song et al., 2021). However, both meta-analyses showed significant heterogeneity; therefore, the results should be viewed dialectically, and further research is required. Previous studies have shown that Asian Indians have higher rates of body fat and abdominal obesity compared with Caucasians (Consultation WE, 2004; Misra and Khurana, 2009), suggesting that the different pathophysiological characteristics of diabetic patients with different ethnicities may be the reason for the heterogeneity. Moreover, the included studies were mostly cross-sectional studies, and some therapeutic interventions, such as metformin, which may be used in the treatment of NAFLD, also have a protective effect on blood sugar management in diabetes (Feng et al., 2019). Additionally, these could have led to the occurrence and development of the two diseases, which cannot be synchronized at the same time period, possibly resulting in biased results.

Based on our results, DR showed a significant positive correlation with the severity of liver fibrosis in NAFLD patients. The appearance of DR could imply more severe NAFLD, and the risk of DR might also reflect the progression of NAFLD. It should be noted that for NAFLD patients with advanced liver disease, including fibrosis, cirrhosis, and hepatocellular carcinoma, a remarkable correlation between NAFLD and DR has been confirmed (El-Serag et al., 2004). Actually, epidemiological evidence has indicated that the severity of NAFLD is closely associated with the risk of diabetic microvascular complications (Targher et al., 2018). The exact responsible mechanism is unclear, but the common pathogenesis of DR in patients with T2DM and those with NAFLD has been identified, including insulin resistance, metabolic inflammation caused by the disorder of glucose and lipid metabolism, and oxidative stress (Gardner et al., 2011; Capitão and Soares, 2016; Sheka et al., 2020; Marušić et al., 2021). NAFLD can also aggravate systemic insulin resistance and hyperglycemia (Marchesini et al., 2005; Day, 2006; Mccullough, 2006; Targher and Arcaro, 2007), which may, in turn, lead to the progression of retinopathy (Brownlee, 2005; Groop et al., 2005; Marshall and Flyvbjerg, 2006). The possible reason is that hepatic fibrosis can exacerbate hepatic and systemic insulin resistance, promote dyslipidemia, and trigger the synthesis of several pro-inflammatory mediators, which could contribute to the occurrence of chronic vascular complications of diabetes (Targher et al., 2016; Targher et al., 2018; Mantovani et al., 2021). The notion that these potential mediators are significantly higher in the blood vessels of diabetic patients with NAFLD than in patients without NAFLD has also been supported by the conclusions of several studies (Horiuchi, 2002; Alessi et al., 2003; Chalasani et al., 2004; Albano et al., 2005; Brownlee, 2005; Abiru et al., 2006; Holt et al., 2006; Targher, 2006; Targher et al., 2007a; Targher et al., 2007b). Additionally, there is increasing evidence that the gut microbiome is associated with the etiology of insulin resistance, NAFLD, and T2DM (Canfora et al., 2019). A dysregulated microbiome and its metabolites can promote the development and progression of hepatocyte steatosis, inflammation, and fibrosis in NAFLD and DR occurrence (Mouzaki and Loomba, 2019; Liu et al., 2021). In conclusion, there is a strong correlation between DR and advanced NAFLD, implying that DR may serve as a clinical biomarker of NAFLD progression.

In addition to finding a significant positive correlation between DR and liver fibrosis in the subgroup analysis, we also found a strong positive correlation between the T1DM subgroup and NAFLD (OR = 2.96, 95% CI: 1.48–5.94). All three included studies involving patients with T1DM came to the same conclusion (Targher et al., 2010a; Yoneda et al., 2012; Vendhan et al., 2014). In fact, a previous study reported a higher prevalence of T1DM in NAFLD patients, which was associated with a higher prevalence of cardiovascular disease (Conway et al., 2010). There is also a study reporting that the hepatic steatosis index is associated with T1DM complications (Tripolino et al., 2019). A study in rhesus monkeys has found more severe retinopathy in T1DM (Xia et al., 2021). Patients with T1DM develop retinopathy earlier, progress faster, and have more severe clinical manifestations (Kumar et al., 2021). Therefore, the positive association between NAFLD and DR in T1DM seems reasonable. Obesity might be a possible mechanism by which NAFLD appears to be associated with T1DM. Epidemiological study has shown that BMI is increasing in people with T1DM (Conway et al., 2010). Increased prevalence of NAFLD in T1DM has been reported in Western countries (Arai et al., 2008; Targher et al., 2010b). T1DM may develop lipoprotein disturbances (e.g., increased apolipoprotein glycosylation and low-density lipoprotein oxidation) and lead to decreased hepatic very low-density lipoprotein (VLDL) output, leading to NAFLD (Regnell and Lernmark, 2011). These metabolic abnormalities can occur even in people with T1DM who are well-controlled (Vergès, 2009), suggesting that the treatment of NAFLD and diabetes-related complications should not only focus on the management of blood sugar but also on the metabolic regulation of the whole body. The possible link mechanism of NAFLD and DR in T1DM still needs more research to explore.

Noteworthy, there was large heterogeneity in our study; thus, the results should be viewed dialectically, and further research should be conducted. Based on our conclusive analysis that DR showed a significant positive correlation with the degree of liver fibrosis in NAFLD patients, a likely source of heterogeneity was that some NAFLD patients included in the study were not staged but only simply distinguished by the presence or absence of NAFLD. In the early stage of NAFLD, probably because the liver lesions are not serious, the released concentration of related communication molecules is not enough to accumulate in the eye and cause lesions. As fibrosis progresses, the concentration of relevant communication molecules is further upregulated, thereby causing lesions in the eye (Cyr et al., 2020; Yuan et al., 2021). Moreover, compared with non-NAFLD patients, NAFLD patients included in this study were relatively young and had a shorter course of diabetes, which might be one of the reasons for the lower prevalence of diabetes complications. Additionally, NAFLD patients with higher body mass index and insulin resistance may be encouraged to make more intensive lifestyle adjustments, such as dietary control and exercise. As a result, they can achieve similar blood glucose control in a shorter time than people without NAFLD, which might be associated with a lower incidence of diabetes complications (Lv et al., 2013).

There were also some limitations to this meta-analysis. First, there was considerable heterogeneity in this meta-analysis, which might be due to study design, type of diabetes, ethnic differences, and severity of NAFLD. Second, most of the studies included were cross-sectional in design; hence, larger longitudinal studies are needed to confirm the associations. Finally, it should also be emphasized that caution should be taken in interpreting the results of this study, considering remaining confounding factors, including unknown or unmeasured risk factors, as well as the potential selection and information biases.

In conclusion, our study found that DR had a significant correlation with the risk of liver fibrosis in NAFLD patients. Thus, screening for DR, by a simple non-invasive fundus photography, easily practicable in ophthalmic outpatient service, allows the detection of patients with T2DM at risk of advanced liver disease and chronic vascular diabetic complications. Future investigations in large prospective cohort studies and further study of the mechanisms are needed.

## Data Availability

The original contributions presented in the study are included in the article/Supplementary Material, further inquiries can be directed to the corresponding authors.
