# Supplementary material for: The presence of diabetic retinopathy closely associated with the progression of non-alcoholic fatty liver disease: A meta-analysis of observational studies
Source: Front Mol Biosci. 2022 Nov 15;9:1019899. doi: 10.3389/fmolb.2022.1019899 (PMC9706004; doi:10.3389/fmolb.2022.1019899)
Supplement: Supplementary file 1 [file DataSheet1.docx]

Supplementary Material

# Supplementary Figures and Tables

## Supplementary Tables

**Supplementary Table 1**. The search strategy of meta-analysis

| Databases | Search strategy |
| --- | --- |
| PubMed | (((("Retinal Diseases"[Mesh])) OR (((((Disease, Retinal) OR (Diseases, Retinal)) OR (Retinal Disease)) OR (Retinopathy)) ))) AND (((("Non-alcoholic Fatty Liver Disease"[Mesh])) OR (((((((((((Non alcoholic Fatty Liver Disease) OR (NAFLD)) OR (Nonalcoholic Fatty Liver Disease)) OR (Fatty Liver, Nonalcoholic)) OR (Fatty Livers, Nonalcoholic)) OR (Liver, Nonalcoholic Fatty)) OR (Livers, Nonalcoholic Fatty)) OR (Nonalcoholic Fatty Liver)) OR (Nonalcoholic Fatty Livers) ) OR (Nonalcoholic Steatohepatitis)) OR (Nonalcoholic Steatohepatitides)) OR (Steatohepatitides, Nonalcoholic)) OR (Steatohepatitis, Nonalcoholic))))) |
| Embase | ((('nonalcoholic fatty liver'/exp) OR ('non alcholic fatty liver disease':ab,ti) OR ('nafld':ab,ti) OR ('nonalcholic fatty liver disease ':ab,ti) OR ('nonalcoholic steatohepatitis')) AND (('retina disease'/exp) OR ('retinopathy':ab,ti))) |
| Cochrane | ((('Non-alcholic Fatty Liver Disease'[Mesh]) OR (("non-alcoholic fatty liver disease" OR "non-alcoholic fatty liver diseases" OR "non-alcoholic fatty- liver disease" OR “NAFLD”):ti,ab,kw) OR ((“non-alcoholic cirrhoses” OR “non-alcoholic cirrhosis” OR “NASH”):ti,ab,kw) OR ((“steato-hepatitis ”OR “steato-hepatitides” OR “steatohepatitis” OR “fatty liver”):ti,ab,kw)) AND ((“Diabetic Retinopathy”[Mesh]) OR (“Retinal Diseases”[Mesh]) OR ((“ retinopathy" OR "retinal disease" OR "retinal diseases"):ti,ab,kw))) |

**Supplementary Table 2.** meta regression analysis association between non-alcoholic fatty liver disease and the risk of diabetic retinopathy in diabetes mellitus

|  | r | 95%CI | SE | t | P-value | I^2^ (%) |
| --- | --- | --- | --- | --- | --- | --- |
| Age (years) | -0.024 | -0.082-0.034 | 0.027 | 0.91 | 0.383 | 95.05 |
| HbA1c (%) | -0.353 | -1.040-0.333 | 0.315 | -1.12 | 0.284 | 94.92 |
| Duration of DM (years) | 0.192 | -0.001-0.384 | 0.087 | 2.19 | 0.051 | 92.32 |
| BMI (kg/m^2^) | -0.094 | -0.465-0.276 | 0.168 | -0.56 | 0.587 | 95.79 |
| AST (U/L) | -0.001 | -0.216-0,216 | 0.098 | -0.00 | 0.997 | 95.86 |
| ALT (U/L) | -0.001 | -0.123-0.120 | 0.054 | -0.03 | 0.979 | 95.86 |

r, regression coefficient; SE, standard error; I^2^, I-squared; DM, diabetes mellitus; BMI, body mass index; AST, aspartic transaminase; ALT, Alanine transaminase.

## Supplementary Figures


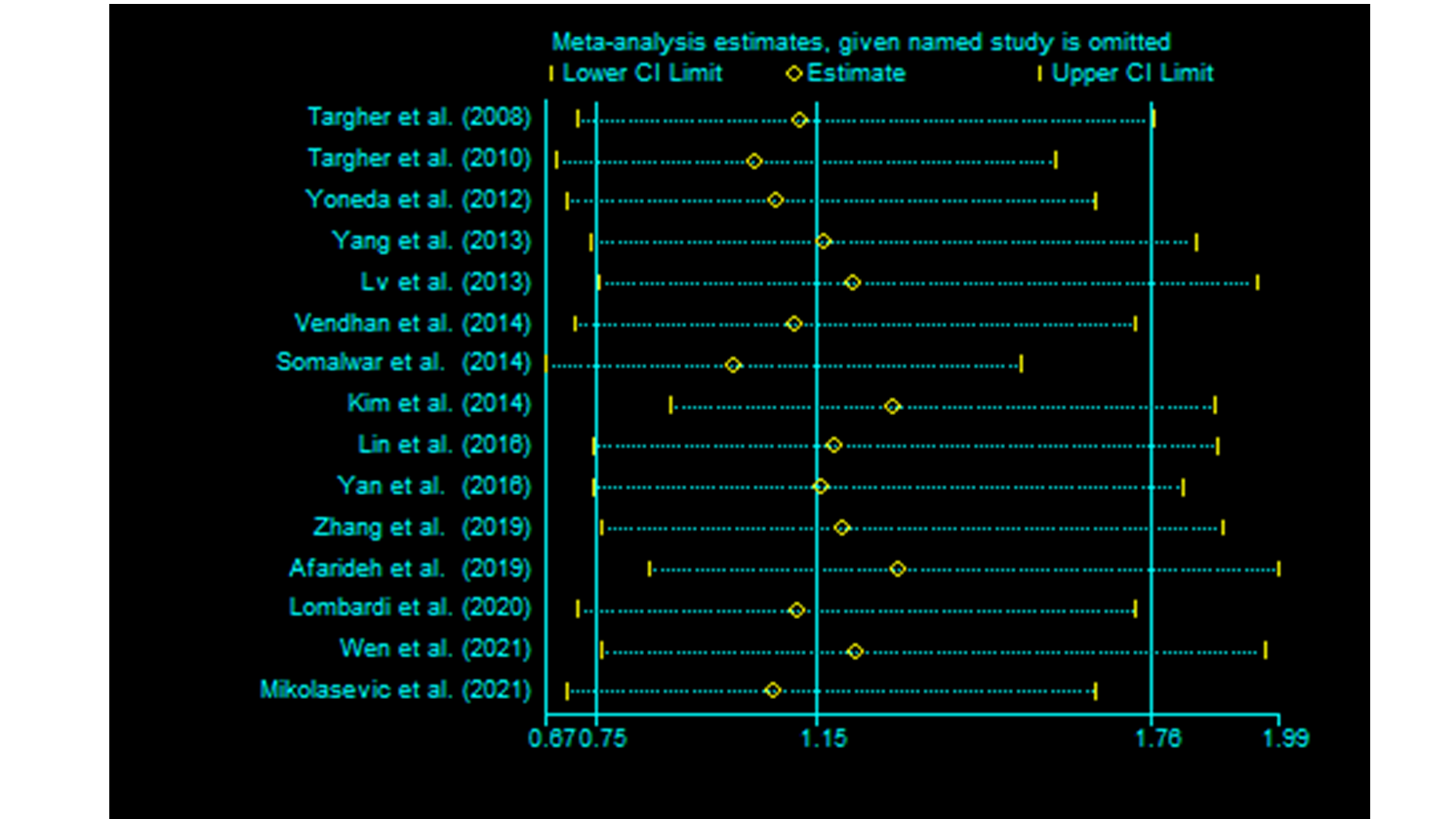


**Supplementary Figure 1**. Meta-analysis of association between non-alcoholic fatty liver disease and the risk of diabetic retinopathy in diabetes mellitus included the effects of single studies. (The vertical line in the middle represents the total combined effect size of mate analysis. The point corresponding to each study represents the combined effect size of the remaining studies after deleting this study)


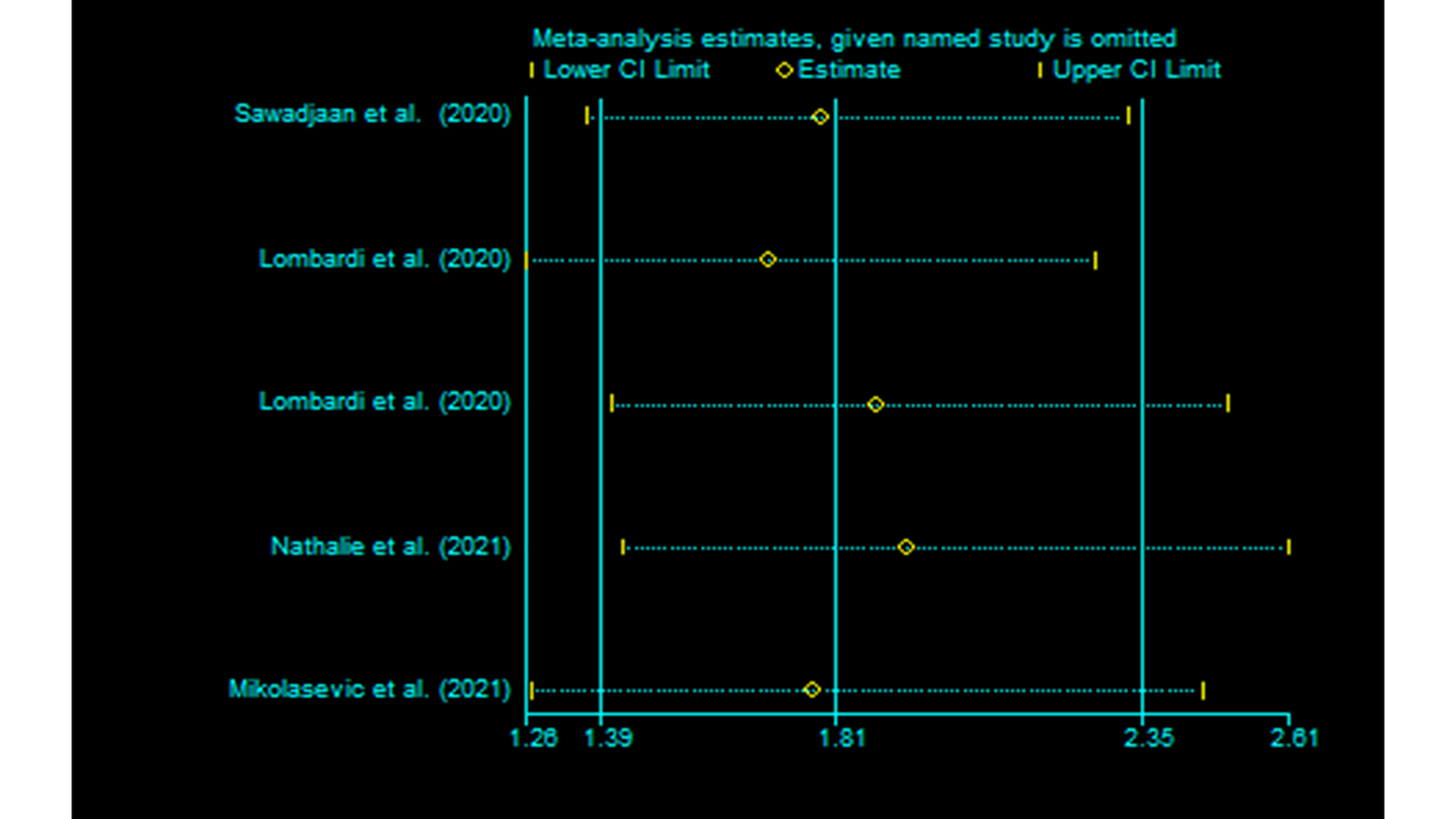


**Supplementary Figure 2**. Meta-analysis of association between liver fibrosis and the risk of diabetic retinopathy in diabetes mellitus included the effects of single studies. (The vertical line in the middle represents the total combined effect size of mate analysis. The point corresponding to each study represents the combined effect size of the remaining studies after deleting this study)


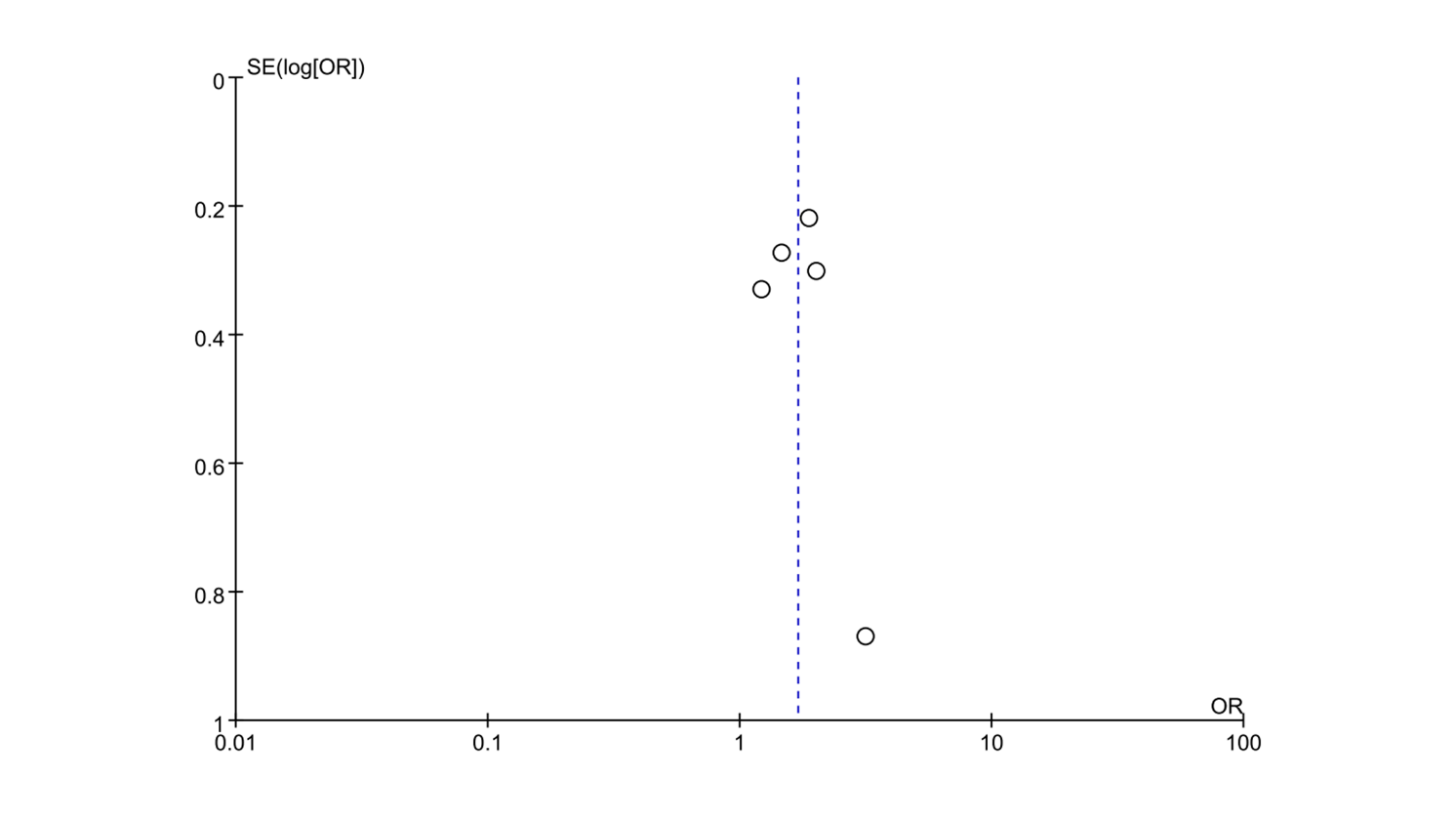


**Supplementary Figure 3**. Funnel plots for meta-analysis of association between liver fibrosis and the risk of diabetic retinopathy in diabetes mellitus.


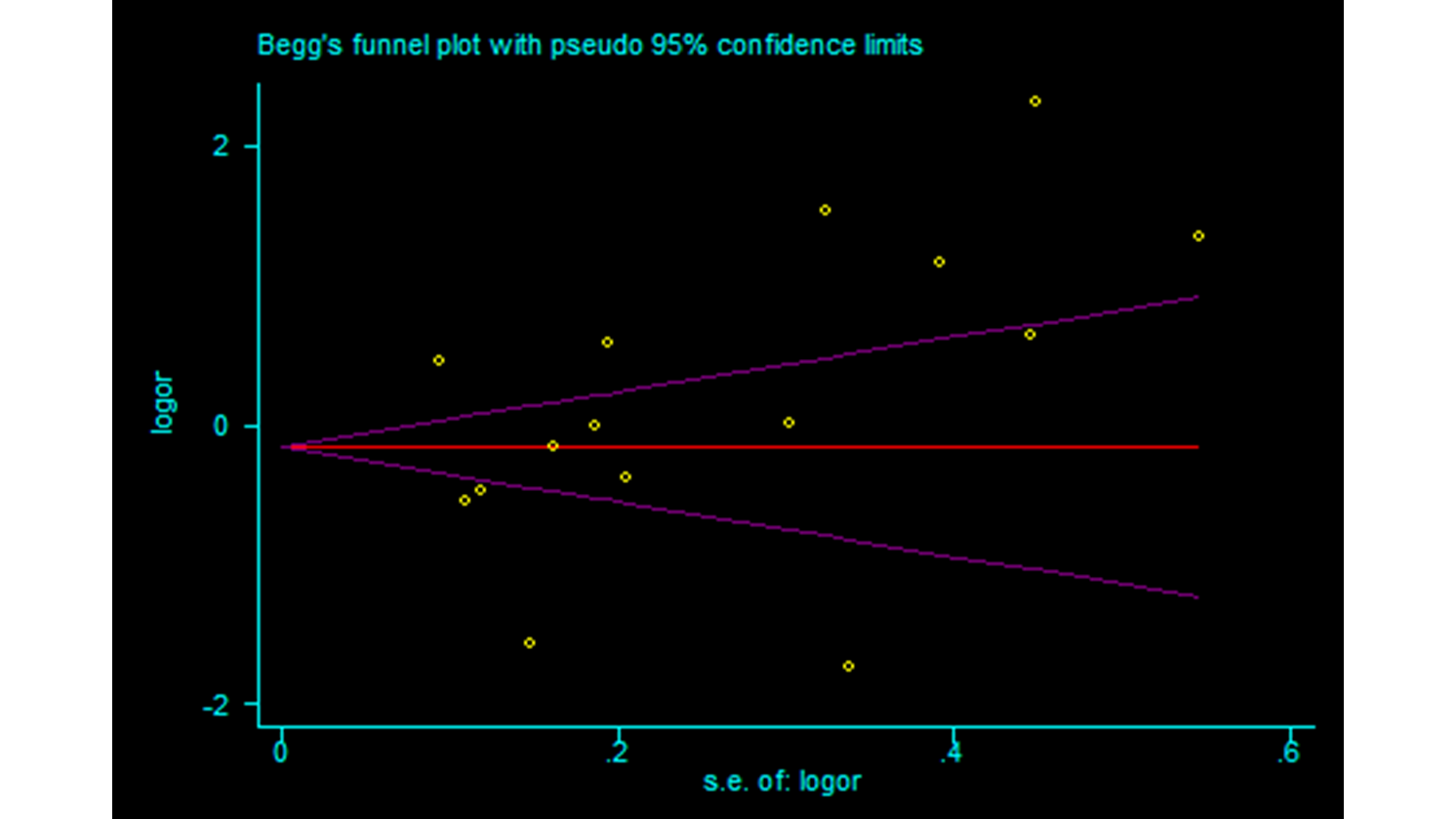


**Supplementary Figure 4**. Begg funnel plots for meta-analysis of association between non-alcoholic fatty liver disease and the risk of diabetic retinopathy in diabetes mellitus. (It is used to evaluate whether there is publication bias)


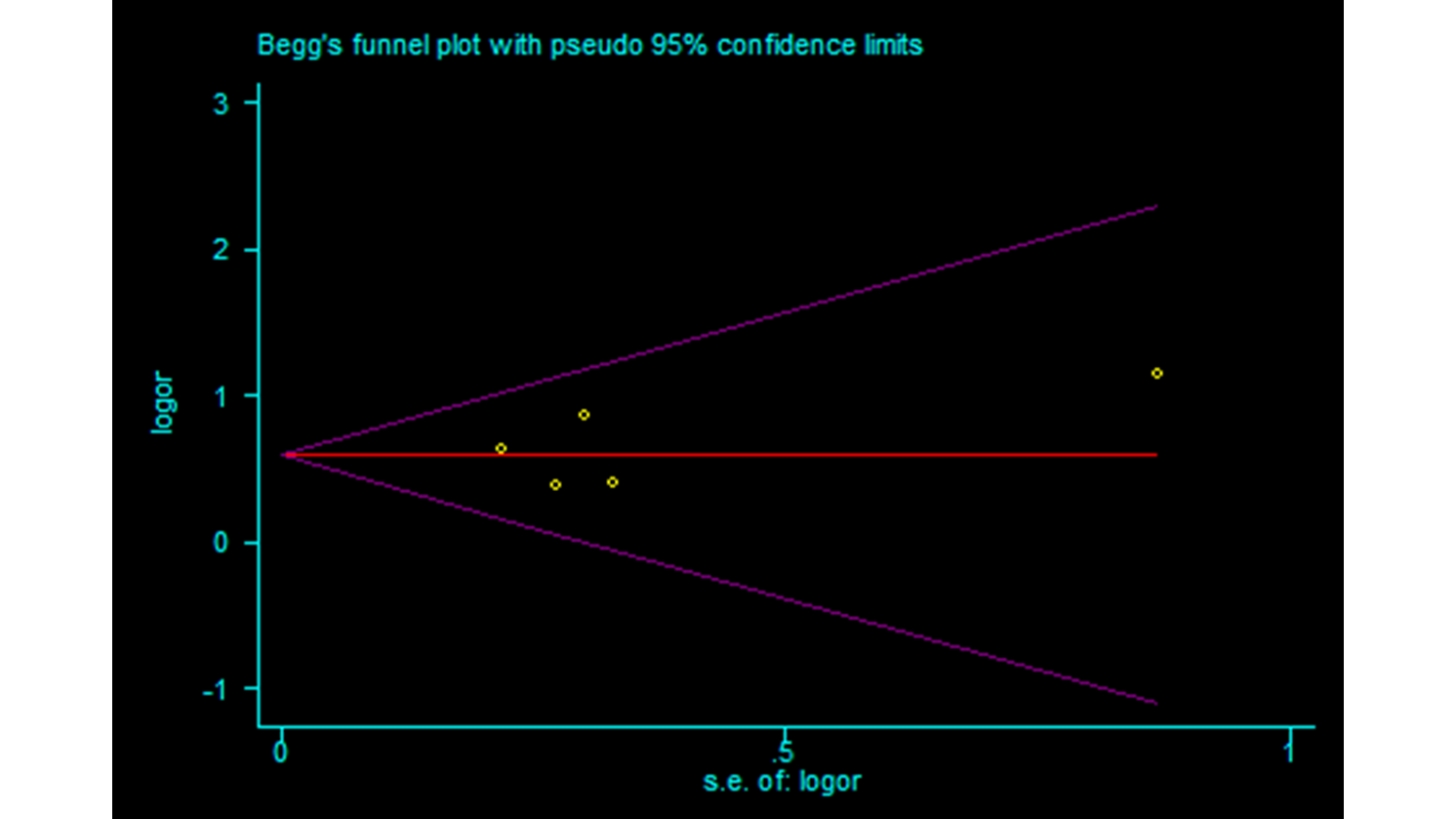


**Supplementary Figure 5**. Begg funnel plots for meta-analysis of association between liver fibrosis and the risk of diabetic retinopathy in diabetes mellitus. (It is used to evaluate whether there is publication bias)
